# Pneumoparotid: A Rare Consequence of Being a Saxophonist

**DOI:** 10.7759/cureus.73497

**Published:** 2024-11-11

**Authors:** João Santos Silva, Henrique Donato, Vasco Mendes, Paulo Donato

**Affiliations:** 1 Radiology, Centro Hospitalar e Universitário de Coimbra, Coimbra, PRT

**Keywords:** cheek swelling, gas, gas insufflation, parotid enlargement, pediatric otolaryngologist, pneumoparotid

## Abstract

A teenage girl who had recently begun playing saxophone presented with recurrent painless right cheek swelling for six months. Despite several courses of antibiotics, the swelling continued to re-occur. On examination, enlargement of the right parotid gland and crepitus over the same region are noted, with no signs of acute inflammation.

Ultrasound examination of the right parotid gland revealed linear areas of increased echogenicity with equivocal posterior shadowing. Computed tomography (CT) showed enlargement of the right parotid gland with numerous gas bubbles in Stensen’s duct and their branches without any signs of inflammation.

Pneumoparotid represents an uncommon etiology of parotid gland enlargement. It is characterized by air infiltration within the parotid duct system and/or gland parenchyma secondary to retrograde air reflux via Stensen’s duct. This condition is often seen in situations generating significant intraoral pressure, including activities such as playing wind instruments.

Diagnosis relies on clinical examination and is confirmed through diagnostic imaging. Treatment is conservative and predominantly involves warm compresses. However, antibiotics may be prescribed to prevent secondary suppurative parotitis.

## Introduction

Pneumoparotid represents an uncommon etiology of parotid gland enlargement, characterized by air infiltration within the parotid duct system and/or gland parenchyma, secondary to retrograde air reflux via Stensen’s duct. As of June 30, 2022, only 170 cases have been documented in the literature [1,2].

The condition was first described by Hyrtl in 1865, and subsequent literature has documented it under various terms, including pneumosialadenitis, pneumoparotitis, wind parotitis, and surgical or anesthetic mumps. While the terms pneumoparotid and pneumoparotitis are often used interchangeably, it is essential to differentiate between them. Pneumoparotid is the more accurate term, referring specifically to the presence of air within the parotid gland without accompanying inflammatory or infectious symptoms [3].

Pneumoparotid arises when elevated intraoral pressure leads to retrograde airflow into Stensen’s duct, often in the context of compromised anatomical barriers such as the mucosal flap or buccinator muscle. This condition is commonly associated with activities that generate high intraoral pressure such as playing wind instruments, dental procedures, or positive pressure ventilation during anesthesia. Psychological factors, including nervous tics or adjustment disorders in adolescents, may also contribute. Imaging, particularly CT with a "puffed-cheek" technique, can induce or reveal this condition [3,4].

## Case presentation

An 11-year-old girl, who had recently started playing the saxophone, presented with a six-month history of recurrent, painless swelling of the right cheek. Despite multiple courses of antibiotics, the swelling persisted. The episodes were unrelated to food intake. The patient denied malaise, a history of fever, or recent dental procedures. Her past medical history was unremarkable.

On examination, enlargement of the right parotid gland and crepitus in the same region were noted, with no signs of acute inflammation. Clear saliva was expressed from both parotid duct orifices, with no apparent narrowing of either orifice. Examination of the neck, nose, ears, and throat revealed no abnormalities.

**Figure 1:**
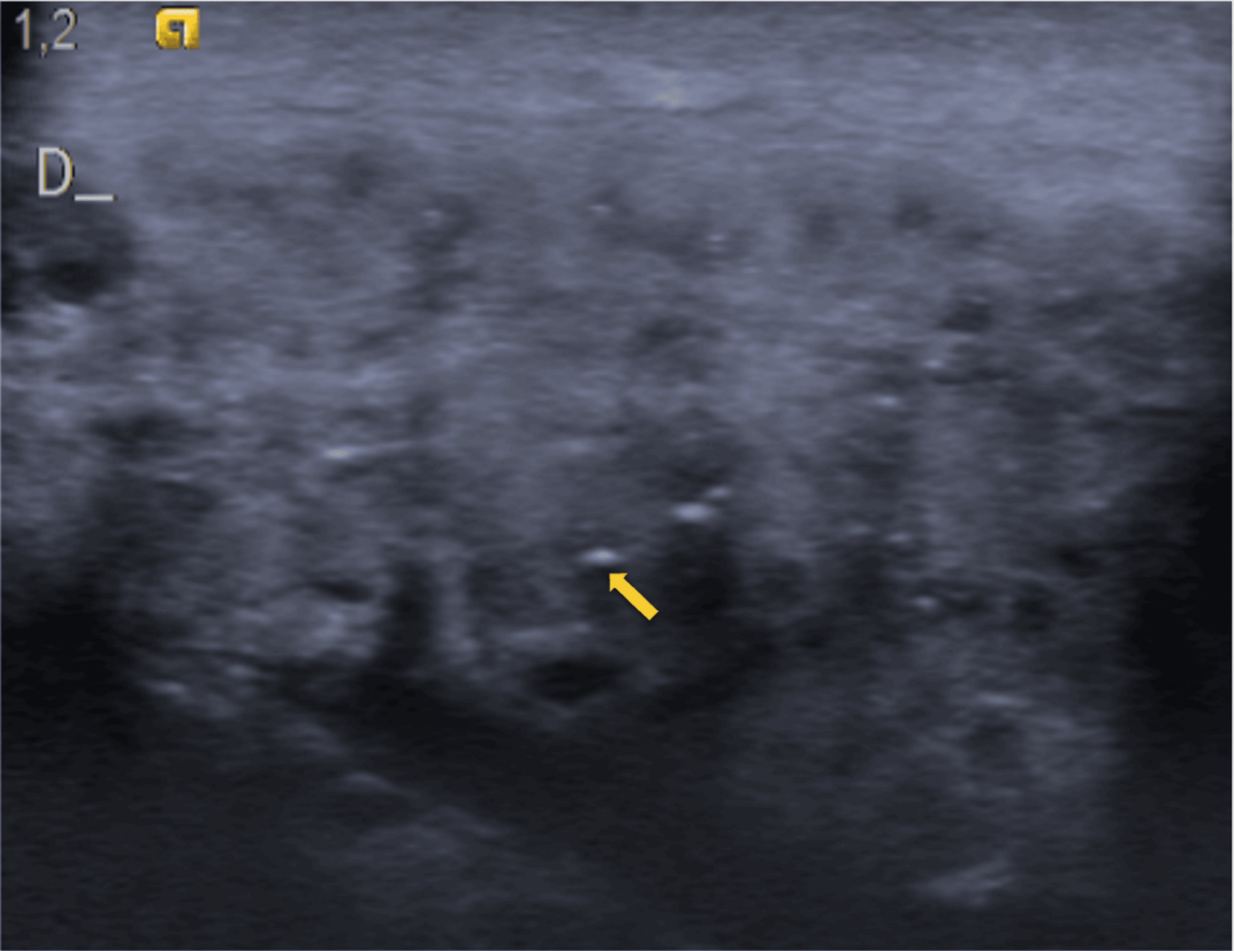
Ultrasound Ultrasound image of the right parotid gland, which appears enlarged and heterogeneous, shows multiple anechoic spaces corresponding to ductal branches, containing echogenic foci (arrow) that represent gas bubbles.

Laboratory tests, including a full blood count, erythrocyte sedimentation rate (ESR), renal and liver function tests, and serum electrolyte levels, were all within normal limits. Ultrasound of the right parotid gland revealed an enlarged and heterogeneous gland, with multiple anechoic spaces containing echogenic foci and no additional findings such as organized collections (Figure 1). 

**Figure 2:**
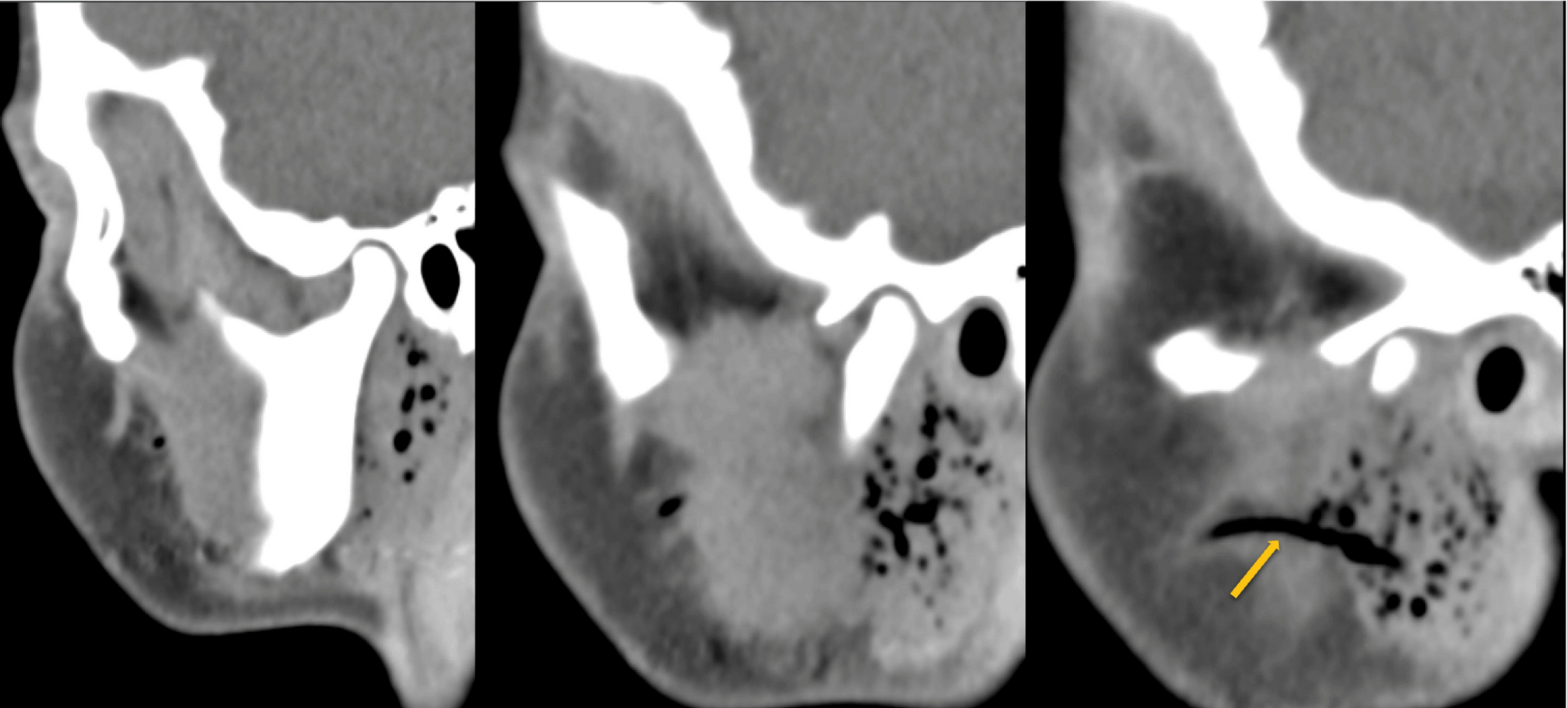
CT scan Sagittal CT sections demonstrate enlargement of the right parotid gland with multiple millimeter-sized intraglandular gas bubbles and no signs of inflammation. Gas is also observed within Stensen’s duct, extending to the level of the buccinator muscle (arrow).

**Figure 3:**
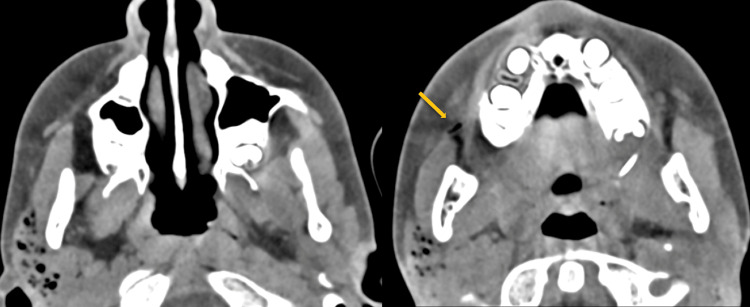
CT scan Axial CT sections demonstrate enlargement of the right parotid gland with multiple millimeter-sized intraglandular gas bubbles and no signs of inflammation. Gas is also observed within Stensen’s duct, extending to the level of the buccinator muscle (arrow).

**Figure 4:**
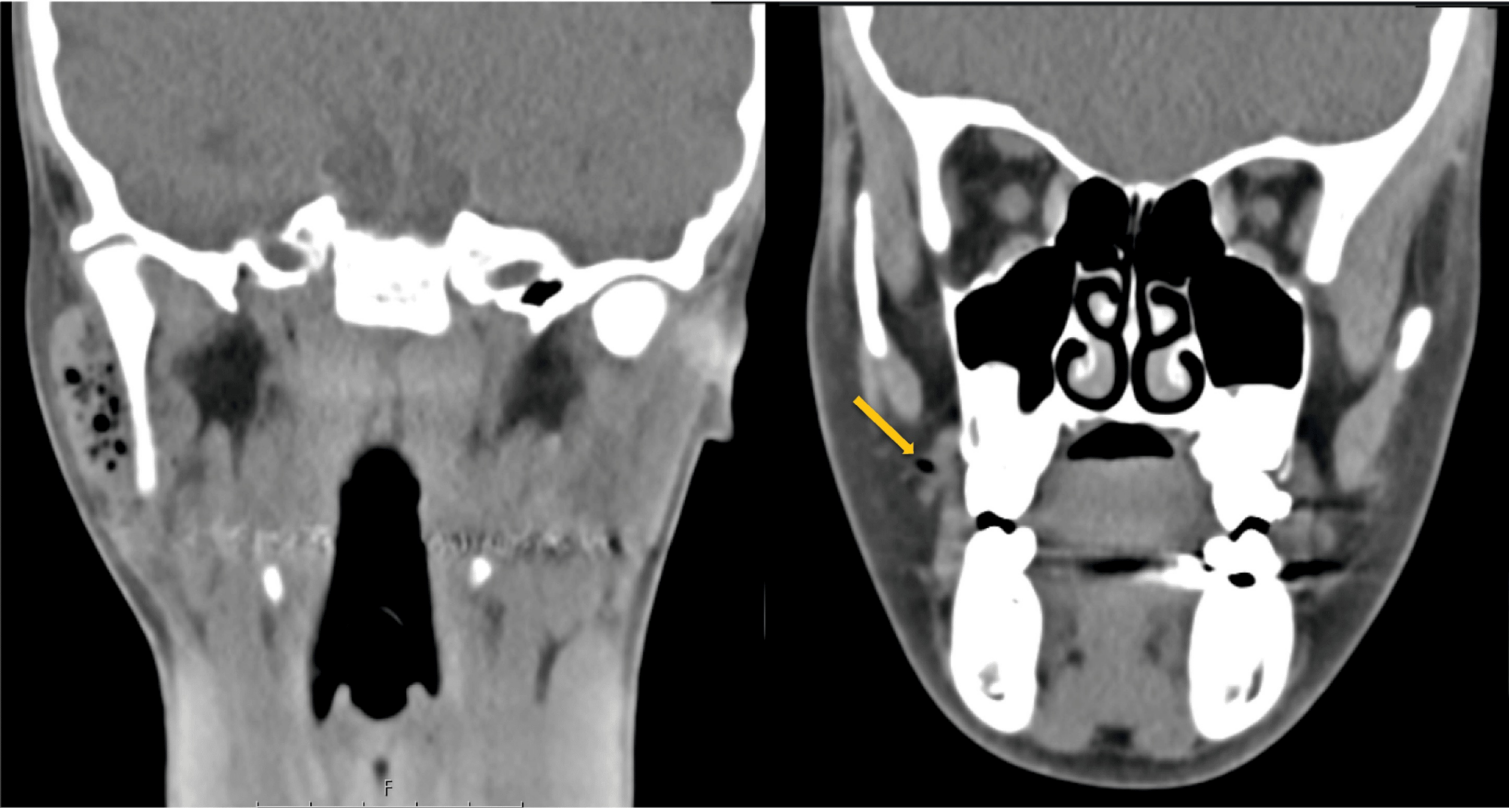
CT scan Coronal CT sections demonstrate enlargement of the right parotid gland with multiple millimeter-sized intraglandular gas bubbles and no signs of inflammation. Gas is also observed within Stensen’s duct, extending to the level of the buccinator muscle (arrow).

Computed tomography (CT) demonstrated enlargement of the right parotid gland with multiple gas bubbles in Stensen’s duct and its branches, without evidence of inflammation. No subcutaneous emphysema was observed. The left parotid gland, as well as bilateral submandibular and sublingual glands, appeared normal with no signs of sialolithiasis (Figures 2-4).

## Discussion

Pneumoparotid is a condition that can occur in individuals such as wind instrument players, glassblowers, and others who experience elevated intraoral pressure, which forces air into the parotid ductal system [4].

Diagnosis relies on clinical examination and imaging studies. Computed tomography (CT) scans provide a definitive assessment, revealing intraglandular gas bubbles of millimeter scale, predominantly located within the ductal system (including the Stensen duct and its branches) in the absence of inflammatory signs. Ultrasound, often the initial imaging modality, typically shows an enlarged gland with multiple echogenic foci, representing gas within anechoic spaces aligned with the ductal system, though diagnosis via this method is more challenging. The absence of sialolithiasis further supports the diagnosis of pneumoparotid [4-8].

Management is primarily conservative, focusing on warm compresses and antibiotics can sometimes be useful to prevent secondary suppurative parotitis. However, in cases where complications such as pneumoparotitis arise, antibiotic therapy becomes essential [3-5].

In rare cases, air may rupture the parotid acini and extend into the parapharyngeal space, causing subcutaneous emphysema. In severe cases, the condition may progress to involve the retropharyngeal space, potentially leading to pneumothorax or pneumomediastinum. Management of complications such as subcutaneous emphysema requires careful evaluation of clinical parameters, size, and recurrence rates to prevent progression to pneumomediastinum. For significant cases, interventions such as needle aspiration or surgical treatment may be warranted. Recurrent pneumoparotid may also necessitate aggressive surgical approaches [3-7].

The patient was diagnosed with pneumoparotid, likely associated with saxophone playing. No evidence of parotid gland infection was noted. Symptoms resolved within a few days following conservative management.

## Conclusions

Pneumoparotid, though rare, is a well-documented clinical condition and should be considered in the differential diagnosis of parotid gland swelling, especially in pediatric and adolescent populations. Pneumoparotid is a non-physiological condition characterized by air within Stensen’s duct and the parotid gland. While generally non-inflammatory, it can lead to complications such as subcutaneous emphysema or, more rarely, pneumothorax or pneumomediastinum.

Early diagnosis is facilitated by CT imaging, and management is primarily conservative.
